# A putative lipase gene *EXTRA GLUME1* regulates both empty-glume fate and spikelet development in rice

**DOI:** 10.1111/j.1365-313X.2008.03710.x

**Published:** 2008-11-07

**Authors:** Haoge Li, Dawei Xue, Zhenyu Gao, Meixian Yan, Wenying Xu, Zhuo Xing, Danian Huang, Qian Qian, Yongbiao Xue

**Affiliations:** 1 The State Key Laboratory of Rice Biology, College of Life Sciences, Zhejiang UniversityHangzhou, 310029 China; 2 Laboratory of Molecular and Developmental Biology, Institute of Genetics and Developmental Biology, Chinese Academy of Sciences and National Center for Plant Gene ResearchBeijing 100101, China; 3 The State Key Laboratory of Rice Biology, China National Rice Research InstituteHangzhou 310006, China; 4 College of Biological Science and Technology, Shenyang Agricultural UniversityShenyang 110161, China

**Keywords:** empty glume, spikelet development, floral meristem, lipase, rice

## Abstract

Recent studies have shown that molecular control of inner floral organ identity appears to be largely conserved between monocots and dicots, but little is known regarding the molecular mechanism underlying development of the monocot outer floral organ, a unique floral structure in grasses. In this study, we report the cloning of the rice *EXTRA GLUME1* (*EG1*) gene, a putative lipase gene that specifies empty-glume fate and floral meristem determinacy. In addition to affecting the identity and number of empty glumes, mutations in *EG1* caused ectopic floral organs to be formed at each organ whorl or in extra ectopic whorls. Iterative glume-like structures or new floral organ primordia were formed in the presumptive region of the carpel, resulting in an indeterminate floral meristem. *EG1* is expressed strongly in inflorescence primordia and weakly in developing floral primordia. We also found that the floral meristem and organ identity gene *OsLHS1* showed altered expression with respect to both pattern and levels in the *eg1* mutant, and is probably responsible for the pleiotropic floral defects in *eg1*. As a putative class III lipase that functionally differs from any known plant lipase, *EG1* reveals a novel pathway that regulates rice empty-glume fate and spikelet development.

## Introduction

The formation of a flower is a complicated process marked by conversion of the identity of the shoot apical meristem to that of an inflorescence meristem, and subsequently forming floral meristems from the lateral margins within a group of cells (Coen and Nugent, 1994). Floral meristems produce flowers that typically consist of four whorls of organs: sepal, petal, stamen and carpel (pistil). In contrast to the indeterminate shoot apical meristem, floral meristems cease cell proliferation after formation of the carpel. During this process, morphogenetic signals within the flower meristem are generated, transmitted, perceived and acted on to generate specific floral organs of appropriate size and shape at fixed locations (Conti and Bradley, 2007; Golz and Hudson, 2002; Griffith *et al.*, 1999; Smyth, 2005).

In the past two decades, a growing body of information has accumulated regarding the molecular genetic pathway of how flowers form and differentiate, especially in two model eudicots, *Arabidopsis thaliana* and *Antirrhinum majus* (Zik and Irish, 2003). The ABC model, which postulates that combinatorial activities of three classes of floral homeotic genes specify floral organ identity, is widely accepted (Coen and Meyerowitz, 1991). Recent studies have shown that *SEPALLATA* (*SEP*) genes are also required for the development of all four whorl organs (Ditta *et al.*, 2004; Pelaz *et al.*, 2000). Compared to organ identity genes, floral meristem identity (FMI) genes may play a more pivotal role in floral organ formation in that they not only prevent young floral meristems from reverting to inflorescences, but also ensure that the floral patterning and differentiation program is initiated in the correct location. Many FMI genes, mainly transcription factors, have been cloned in dicots. In Arabidopsis, *LEAFY* and *APETALA1* repress expression of *TERMINAL FLOWER1* (*TFL1*) and *AGAMOUS-LIKE24* (*AGL24*) in floral meristems (Krizek and Fletcher, 2005; Liljegren *et al.*, 1999; Ratcliffe *et al.*, 1999; Yu *et al.*, 2004). *WUSCHEL* (*WUS*) is required for maintaining the proliferative capacity of floral meristems, and is expressed in a subset of floral meristem cells (Schoof *et al.*, 2000). The repression of *WUS* by *AGAMOUS* (*AG*) terminates meristematic activity to allow a floral meristem to differentiate into carpel primordia (Lenhard *et al.*, 2001; Lohmann *et al.*, 2001).

In contrast to the wealth of information on the molecular regulation of flower development in eudicots, the genetic control of flower development in monocot grasses is far from clear. The structure of a grass flower has features that are distinct from those of eudicots because of its characteristic floral organs (lemma, palea and lodicules) and associated organs (glumes), which, together with stamens and pistils, form the spikelet. The characteristic organs of a spikelet display great diversity even among grasses. Two highly reduced leaf-like rudimentary glumes of rice are produced by a spikelet meristem in a distichous arrangement, and then two empty glumes form opposite to each other and slightly above the rudimentary glumes, which are considered to be vestiges of two lower florets. After producing these glumes, the rice spikelet is converted to a floret meristem, and forms a single floret consisting of one lemma, one palea, two lodicules, six stamens and one pistil (Bommert *et al.*, 2005; Itoh *et al.*, 2005). The glumes of *Eleusine indica* have the same shape and texture as the lemmas (Reinheimer *et al.*, 2006). Maize has two glumes whose lengths reach the apex of the florets, and the *TEOSINTE GLUME ARCHITECTURE1* (*TGA1*) gene is responsible for the differences in glume cell components between *Zea mays* and its ancestor teosinte (Wang *et al.*, 2005). Due to its specific and different morphology and the lack of studies on relevant mutants, the grass spikelet, especially its unique floral organs, has been the center of some controversy (Clifford, 1987; Zanis, 2007). The glumes are generally interpreted as bracts (Clifford, 1987). However, the diversity in the morphology and position of glumes across the grasses have led to uncertainty as to their identity, and our knowledge concerning the molecular control of glume identity and evolution is currently very limited (Zanis, 2007).

Recent studies on transcription factors have revealed that genetic control of inner floral organs appears to be conserved between dicots and grasses, at least to some extent (Itoh *et al.*, 2005). A mis-sense mutation in the lemma and palea identity gene *OsLHS1*, a member of the *SEPALLATA* MADS box genes, caused elongated lemmas, leafy palea/lemma-like organs, reduced stamens and increased carpel numbers (Jeon *et al.*, 2000). The gene *OsSNB*, which controls the transition of spikelet meristem (Lee *et al.*, 2007), and the *UFO*-orthologous gene *OsAPO1* have also been isolated (Ikeda *et al.*, 2005,^2007^). However, upstream genes that regulate the floral organ identity genes have been reported only rarely in grasses (Ikeda *et al.*, 2007). Thus, identification of the pathway that regulates spikelet development beyond these described transcription factors and the genes specifying the characteristic organs in grasses will be informative in understanding the genetic frameworks of grass spikelet formation and evolution.

In this study, we report the isolation of two *extra glume1* (*eg1*) mutant alleles, which produced extra glume-like structures in the spikelet, loss of floral meristem determinacy and a pleiotropic defect in floral organ formation. We determined that *EG1* is involved in empty-glume specification and identified it by positional cloning. Our results demonstrate that *EG1* encodes a putative lipase gene. However, the Arabidopsis gene most closely related to it did not cause any defects in flower development (Padham *et al.*, 2007), suggesting possible functional divergence between these putative lipases. We also revealed that *EG1* regulates floral meristem and floral organ identity by mediating expression of the floral homeotic gene *OsLHS1*. Thus, *EG1* appears to be a component of a potential lipid-signaling pathway controlling rice spikelet development.

## Results

### *eg1* affects both spikelet development and floral meristem determinacy

**Figure 1:**
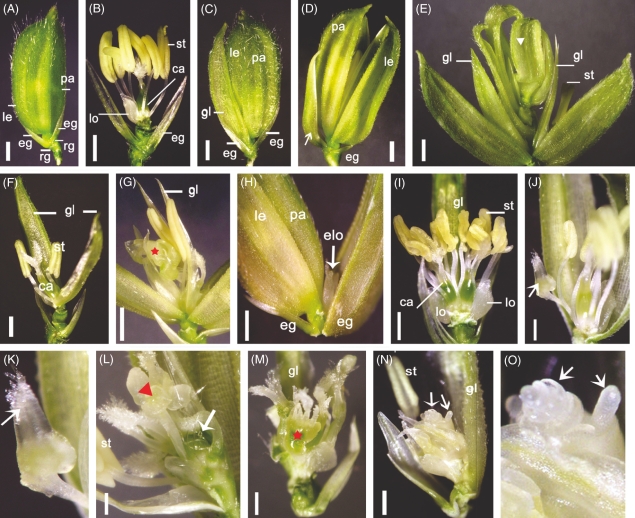
Phenotypes of the *eg1* mutant. (A) Wild-type flower. (B) Wild-type flower with two empty glumes, two lodicules, six stamens and one carpel. The lemma and palea were removed for clarity. (C)–(O) *eg1-1* flowers. The lemma and palea were removed in (F) and (I)–(O) for clarity. (C) *eg1-1* flower. One ectopic glume-like organ is formed. (D) An elongating empty glume (arrow) is shown in an *eg1-1* open-hull flower. (E) *eg1-1* flower with a severe abnormality. The carpel is completely replaced by reiterative glume-like structures (triangle). (F) Glume-like organs are formed in an *eg1-1* flower, with no apparent lodicule. (G) *eg1-1* flower with a fleshy organ of uncertain identity (asterisk) and lodicules transformed to glume-like organs. (H) Lodicules are ectopically formed between the palea and an elongating empty glume in an *eg1-1* flower (arrow). (I) A twin *eg1-1* flower. The inner three floral organs are doubled. (J) One stamen is transformed into a stigma-like organ (arrow) in an *eg1-1* flower. (K) Partial magnification of (J). (L) Ectopic fleshy cell mass (arrowhead) and filamentous structure (arrow) emerging from a carpel-like organ in an *eg1-1* flower. (M) *eg1-1* flower showing multiple fused-carpels (asterisk), with 11 stigmas. (N) *eg1-1* flower in which differentiated primordia have taken the place of the carpel, showing a developmental inversion (arrow). (O) Partial magnification of (M). ca, carpel; eg, empty glume; elo, ectopic lodicule; gl, glume-like organ; le, lemma; lo, lodicule; pa, palea; rg, rudimentary glume; st, stamen. Scale bars = 1 mm (A, C–E), 100 μm (B, F–I, M) or 50 μm (J, L, N).

A wild-type rice floret typically consists of one lemma, one palea, two lodicules at the lemma side in whorl 2, six stamens in whorl 3, and one carpel with a pair of white stigmas and a green ovary in whorl 4. A floret together with two pairs of sterile glumes (rudimentary glumes and empty glumes), which subtend at its base, constitute a spikelet (Figure 1A,B).

The rice mutant *extra glume1* (*eg1*) has been described previously and named on the basis of an extra glume-like structure between palea and lemma (Iwata and Omura, 1971). We have isolated two new *eg1* mutant alleles (*eg1-1* and *eg1-2*) that showed no apparent difference from wild-type plants in the vegetative phase or the number of panicles (data not shown), but exhibited a wide variety of spikelet developmental defects from outer to inner floral organs except for the rudimentary glumes. The abnormities included the occurrence of glume-like organs, alteration of the patterning and number of floral organs, and, in some extreme cases, loss of flower determinacy. *eg1-2* is a weak allele, for which only the empty glumes, lodicules and stamens are affected. In some flowers, the number of empty glumes increased to three or four, while other floral organs appeared normal. Loss of flower determinacy was very rarely observed in *eg1-2* (Table 1). In contrast, *eg1-1* is a strong allele. In addition to the floral defects seen in *eg1-2*, additional florets were produced in the central region in *eg1-1* mutants, and floral patterning (identity and position) defects occurred in nearly in all whorls (Table 1). As the *eg1-1* mutant allele had all the representative defects exhibited in *eg1-2*, we selected *eg1-1* for detailed phenotypic studies.

**Table 1:** Floral organ number (mean ± SEM) in wild-type and *eg1* plants

Genotype	Number of flowers examined	Gl^1^^a^	Lemma/palea	Lodicules	Stamens	Pistils	Gl^2^^b^	Additional florets
Wild-type	100	0.00 ± 0.00	2.00 ± 0.00	2.00 ± 0.00	6.00 ± 0.00	1.00 ± 0.00	0.00 ± 0.00	0.00 ± 0.00
*eg1-1*	100	1.20 ± 1.71	2.00 ± 0.00	0.93 ± 1.36	2.27 ± 2.35	0.76 ± 0.87	1.03 ± 2.27	0.47 ± 0.86
*eg1-2*	100	0.90 ± 0.40	2.00 ± 0.00	1.80 ± 0.61	5.90 ± 0.40	1.00 ± 0.00	0.20 ± 0.61	0.00 ± 0.00

a Gl^1^, glume-like organs outside the lemma.

b Gl^2^, glume-like organs inside the palea.

A glume-like organ formed in a new whorl between empty glumes and the lemma in the *eg1-1* mutant (Figure 1C), or developed from an apparent homeotic transformation of one or two empty glumes (Figure 1D). Such organs also formed inside the palea, along or across the axis of the lemma and palea (Figure 1E–G). Approximately 10% of *eg1-1* flowers had irregularly shaped palea and lemma, resulting in open-hull flowers (Figure 1D). The lodicules of the *eg1-1* flowers were also affected by an apparent homeotic transformation to glume-like organs (Figure 1G) or formed ectopically outside the lemma and palea (Figure 1H). Occasionally, the number of lodicules was also affected, varying from 0 to 4 (Figure 1F,I and Table 1). The most prominent phenotype of *eg1-1* flowers occurred in the inner two whorls. Variations in stamen number were common in the third whorl. The number of stamens varied from 1 to 12 in the *eg1-1* flowers examined (Figure 1E,I and Table 1). Rarely, a partial homeotic transformation was also observed in the third whorl. Mosaics of stamen and carpel tissues were characterized by being tipped with a stigma and based with a filament (Figure 1J,K). Interestingly, an undifferentiated fleshy cell mass and filamentous structure that had no clear wild-type counterpart emerged from carpel-like organs (Figure 1G,L). An increase in carpel number was also observed in the *eg1-1* flowers (Table 1). Two separate carpels together with four lodicules and 12 stamens formed a twin flower (Figure 1I). More often, multiple carpels were fused together with enlarged stigmas (Figure 1M). Changes in organ number are often associated with a change in meristem size (Clark *et al.*, 1997; Suzaki *et al.*, 2004). Taken as a whole, however, there was no significant difference in the size of floral and apical meristems between *eg1-1* mutant and wild-type plants (data not shown), indicating that the number of *eg1-1* floral organs increased in some flowers but decreased in others. Conspicuously, indeterminate development of floral organs occurred in severe *eg1-1* flowers: some florets reiterated a set of floral organs consisting of the glume-like organs and 1–3 stamens in place of carpels (Figure 1E). On rare occasions, differentiated floral primordia were produced in the central region of a nearly mature *eg1-1* flower (Figure 1N,O). Taken together, these phenotypic alterations suggest that floral meristem determinacy was lost or reversed in some *eg1-1* flowers. We also observed that in both *eg1-1* and *eg1-2* plants, a few flowers similar to the wild-type occasionally developed, suggesting that the mutations could be influenced by unknown developmental and/or environmental conditions, although this requires additional study.

### *eg1* affects the structures of floral organs and spikelet development at an early stage

**Figure 2:**
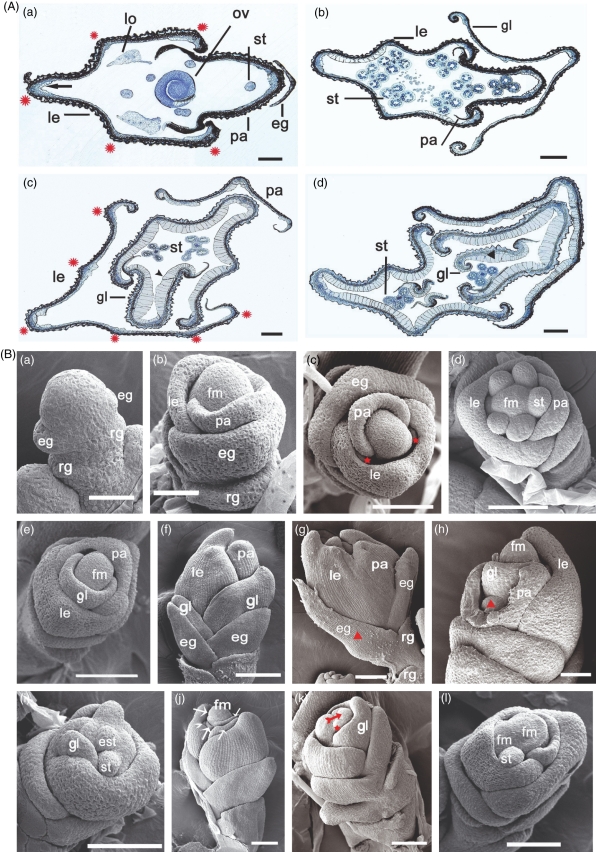
Micrographs of wild-type and *eg1-1* flowers. (A) Histological analysis of wild-type and *eg1-1* flowers. (a) Transverse section of a wild-type spikelet showing five vascular bundles of lemma (star) and inner epidermis cells (arrow). (b) *eg1-1* flower comprising seven stamens and one glume-like organ that have similar cell type to wild-type. (c) Inner epidermis cells (arrowhead) and six vascular bundles of lemma (star) in an *eg1-1* flower. (d) Severe abnormality of an *eg1-1* flower with more glume-like organs and enlarged inner epidermis cells (triangle). Scale bars = 200 μm. (B) Scanning electron micrographs of wild-type and *eg1-1* flowers. (a)–(d) Wild-type flowers. (a) Emerged empty-glume primordia. (b) Formation of lemma and palea primordia. (c) Wild-type flower with lodicule primordia initiated at the lemma side (asterisk). (d) Wild-type flower with six stamen primordia and a flat floral meristem. (e)–(l) *eg1-1* flowers. (e) Formation of a glume-like organ at the lemma side. (f) Formation of a new whorl consisting of two glume-like organs outside the lemma and palea. (g) *eg1-1* flower with an elongated empty glume (triangle) that is similar to the lemma. (h) *eg1-1* flower in which a lodicule has formed ectopically at the palea side (triangle). (i) *eg1-1* flower producing a stamen primordium ectopically in the center of the floral meristem. (j) *eg1-1* flower in which multiple glume-like organs have formed (arrow), and the floral meristem remains bulged at the late stage instead of being flat. (k) The floral meristem is uneven and bifurcated (arrow and star). (l) Formation of a twin floral meristem. eg, empty glume; est, ectopic stamen; fm, floral meristem; gl, glume-like organ; le, lemma; ov, ovary; pa, palea; rg, rudimentary glume; st, stamen. Scale bars = 200 μm (d, h, l) or 50 μm otherwise.

The abnormalities exhibited from the outer whorls to inner whorls in *eg1-1* flowers were clearly revealed by histological analyses. The glume-like organ that formed outside the lemma had similar cell types to those of wild-type palea and lemma (Figure 2Aa,b), whereas such glume-like organs that developed inside the palea often had more and larger inner epidermis cells than wild-type palea and lemma (Figure 2Ac,d). The shape of the palea and lemma in the *eg1-1* flowers were also affected. In contrast to the hooked-hull locked by the five-vascular-bundle lemma and three-vascular-bundle palea in the wild-type (Figure 2Aa), some of the lemma in *eg1-1* mutants had six vascular bundles and showed a different angle to the palea, leaving the flower open-hull (Figure 2Ac), hence *eg1-1* also affected the differentiation of lemma and palea.

To further examine the early developmental defects, we observed *eg1-1* mutant flowers in detail via scanning electron microscopy (SEM). *eg1-1* flower development proceeded normally until emergence of the empty glume primordia. In wild-type flowers, after differentiating a pair of rudimentary glumes and a pair of empty glumes, the spikelet meristem was converted into a floret meristem to produce one lemma and one palea (Figure 2Ba,b). In contrast, in *eg1-1* flowers, a glume-like organ could be observed on the lemma side of the meristem (Figure 2Be) or developed between empty glumes and lemma, suggesting that an additional whorl had formed (Figure 2Bf). In some *eg1-1* flowers, empty glumes had a trichome surface similar to that of a wild-type lemma and elongated almost to the length of the lemma, suggesting that the empty glumes were at least partially transformed into lemma (Figure 2Bg). As development progressed, more striking and multiple flower abnormalities were observed in the inner whorls of *eg1-1* flowers. In wild-type flowers, two lodicules were positioned on the lemma side (Figure 2Bc). In *eg1-1*, ectopic lodicules formed near the base of the palea (Figure 2Bh). Six stamen primordia were produced in a concentric whorl in wild-type flowers (Figure 2Bd), but, in *eg1-1*, in addition to stamens at the flank of floral meristem, ectopic stamen were produced in the central region of the floral meristem (Figure 2Bi), suggesting mis-positioning of stamen primordia.

Floral meristem determinacy was also markedly affected by *eg1-1* mutations. In wild-type flowers, the floral meristem at the stage of stamen initiation tended to be flat (Figure 2Bd) (Itoh *et al.*, 2005). In *eg1-1* flowers, however, even after the palea and lemma had elongated greatly and multiple glume-like organs had been produced, the floral meristem still bulged (Figure 2Bj), as seen at the stage of empty-glume initiation in the wild-type (Figure 2Ba), indicating that the floral meristem had the potential to differentiate more glume-like structures and become indeterminate. Interestingly, in other *eg1-1* flowers, the uneven and bifurcated floral meristem indicated an affected meristem identity (Figure 2Bk). Such a meristem could result in distorted floral primordia and produce structures that had no clear wild-type counterpart. Occasionally, a doublet floral meristem was formed in *eg1-1* flowers (Figure 2Bl), which is likely to produce two sets of inner floral organs, in accordance with the phenotypes we observed in mature *eg1* flowers (Figure 1H). Taken together, these results confirm that early developmental defects, including the formation of ectopic floral organs, changes in organ number and alteration of floral meristems, occurred in *eg1-1* flowers.

### Molecular cloning of *EG1*

**Figure 3:**
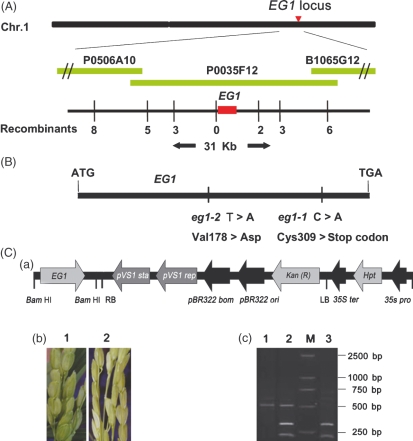
Positional cloning of *EG1*. (A) Fine mapping of the *EG1* locus. The region of the *EG1* locus was narrowed to a 31 kb region on chromosome 1 that contained two predicted open reading frames. (B) Schematic structure of *EG1*. The intronless *EG1* gene was predicted to encode a putative lipase. Two mutant alleles of the *EG1* gene contained base substitutions that produce a stop codon in *eg1-1* or cause amino acid exchange in *eg1-2*. (C) Complementation test. (a) Schematic structure of the complementation construct pCAMBIA1300-EG1. (b) The defects in *eg1-2* flowers were completely rescued by introduction of pCAMBIA1300-EG1. 1, *eg1-2*; 2, the transformed rice line. (c) Identification of transgenic *eg1-2* plants. The V→A substitution in the *eg1-2* allele generates a *Bsm*AI site. M, DNA molecular markers; lane 1, *eg1-2*; lane 2, transformed rice line 1; lane 3, Zhonghua11 (wild-type).

To elucidate the molecular function of *EG1*, we used a positional cloning strategy to identify the *EG1* gene. The *EG1* locus was previously mapped to a physical length of approximately 400 kb on the long arm of rice chromosome 1 (Van Houten *et al.*, 1996; Yoshimura *et al.*, 1997). By using 713 F_2_ plants derived from a cross between *eg1-1* and the wild-type rice *indica* variety ZF802, the *EG1* locus was further delimited to a 2.4 cM region between the SSR markers RM1361 and RM3482. By using newly developed SSR, STS and CAPS markers, we further confined the *EG1* locus to a 31 kb region in PAC clone P0035F12 (Figure 3A). This region was predicted to contain two genes using the rice genome automated annotation system (RGAAS, http://ricegaas.dna.affrc.go.jp). Genome sequencing indicated that both *eg1* alleles had single base-pair substitutions in a predicted putative intronless lipase gene designated *P0035F12.11* (Figure 3B). The single base substitutions of C to A in *eg1-1* and T to A in *eg 1-2* resulted in mutation of Cys309 to a stop codon in *eg 1-1* and Val178 to Asp178 in *eg 1-2* (Figure 3B). To verify whether this putative lipase gene encoded *EG1*, plasmid pCAMBIA1300-EG1 (Figure 3Ca), containing the genomic sequence of the candidate lipase coding region as well as 804 bp upstream and 3506 bp downstream regions, was introduced into *eg1-2* homozygous plants. Two independent transgenic lines were obtained, which showed complete complementation of the *eg1-2* phenotype (Figure 3Cb). In addition, complementation was confirmed as resulting from the introduced wild-type putative lipase gene because the single base-pair substitution in the *eg1-2* produced an additional *Bsm*AI site, which was used as a CAPS marker to discriminate the transformed and untransformed plants (Figure 3Cc). Thus, it can be concluded that the candidate gene *P0035F12.11* does indeed encode *EG1.*

### *EG1* encodes a novel putative triacylglycerol lipase protein

**Figure 4:**
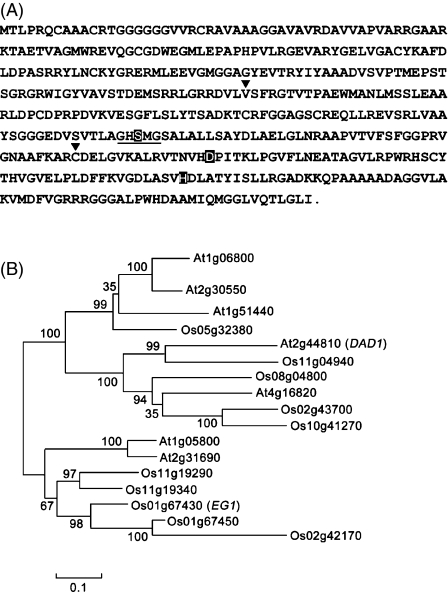
Sequence analysis of the *EG1* gene. (A) Deduced amino acid sequences of EG1. The catalytic triad comprising serine (S), aspartic acid (D) and histidine (H) residues is highlighted. The lipase consensus sequence is underlined. The triangles indicate the amino acids that are substituted in the two *eg1* mutant alleles. (B) Phylogenetic tree derived from alignment of full-length amino acid sequences of EG1 and its most related genes from Arabidopsis and rice using Clustal W together with the neighbor-joining method. Bootstrap values were calculated from 1000 replicates and are given at branch nodes. The scale bar represents 0.1 amino acid substitutions per residue.

Comparison of genomic and cDNA sequences showed that *EG1* has no introns and encodes a putative triacylglycerol (TAG) lipase of 435 amino acids (Figure 4A). TAG lipases, a group of lipolytic enzymes that hydrolyze ester linkages of triglycerides, are widely distributed in animals, plants and prokaryotes, and have been grouped into three classes (Ishiguro *et al.*, 2001). Of these, class III lipases are not closely related to the class I and II lipase families, and a function for this kind of lipase has not yet been reported (Ishiguro *et al.*, 2001; Ryu, 2004). As a predicted class III lipase, EG1 contains a GHSMG motif similar to the lipase consensus sequence (GXSXG), and the position of the putative catalytic triad S266 (within the GHSMG motif), D324 and H371 is identical to that of the catalytic triad of typical fungal and animal lipases (Brady *et al.*, 1990; Winkler *et al.*, 1990; Woolley and Petersen, 1994) (Figure 4A).

To examine the relationship between EG1 and other plant TAG members, we constructed a phylogenetic tree of TAG lipases in Arabidopsis and rice by the neighbor-joining method based on amino acid identities, and found that EG1 is located in a small clade distinct from that of *AtDAD1* (At2g44810) (Figure 4B), a class I Arabidopsis lipase gene sharing 40% amino acid identity with *EG1*. *AtDAD1* is required for pollen maturation, anther dehiscence and flower opening (Ishiguro *et al.*, 2001). The lipase gene that shares the highest homology (57% of amino acid identity) with *EG1* in Arabidopsis is At2g31690 (Figure 4B). However, surprisingly, rather than exhibiting floral organ abnormalities, antisense transgenic plants of this lipase gene were severely stunted and showed delayed rosette senescence (Padham *et al.*, 2007). Thus, the phylogenetic analysis and amino acid comparison suggest that *EG1* is a novel class III lipase gene that is functionally different from any known putative lipase gene in plants.

### Temporal and spatial expression patterns of *EG1*

**Figure 5:**
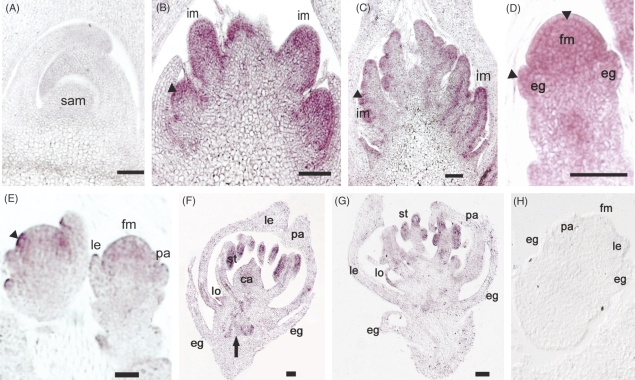
*In situ* localization of *EG1* transcripts in wild-type and *eg1-1* flowers. (A)–(F) Wild-type flowers. (A) Shoot apical meristem prior to reproductive transition. No hybridization signal was detected. (B) *EG1* transcripts (triangle) were detected in the inflorescence meristem at the primary branch stage. (C) *EG1* expression (triangle) was detected in the inflorescence meristem at the secondary branch stage. (D, E) *EG1* transcripts (triangle) are distributed throughout the floral meristems, especially in the newly emerged primordia. (F) *EG1* is expressed weakly in four whorls and the central zone of a nearly mature flower (arrow). (G) *eg1-1* mutant flower. (H) Hybridization with a sense probe. Scale bars = 20 μm (A–D) or 50 μm (E–G).

To gain more insight into the function of *EG1*, we examined the spatial and temporal expression patterns of *EG1* by quantitative real-time PCR and RNA *in situ* hybridization. Very low expression was observed in developing seed and stem, and *EG1* was primarily expressed in young flowers, inflorescence, leaf and root tissues (Figure S1). *EG1* transcripts were not detected in the vegetative shoot apical meristem (Figure 5A), but when the shoot apical meristem converted to an inflorescence meristem, *EG1* transcripts were strongly expressed in the primary and secondary rachis branch meristems (Figure 5B,C). When initiation of the floral organ primordia began, *EG1* was expressed in the developing floral organ primordia (Figure 5D). Expression of *EG1* was also clearly observed in floral meristems, especially in the domain where new floral organ primordia are assumed to have arisen (Figure 5D,E). With ensuing floral development, expression of *EG1* was slightly reduced in the primordia of floral organs. In nearly mature flowers, only a very weak signal was detected in the primordia of stamens, carpel, lodicules, lower parts of the lemma and palea, and the inner central zone of flowers (Figure 5F). *EG1* expression was also examined in the *eg1-1* mutant. A similar pattern of *EG1* expression was seen in the *eg1-1* mutant (Figure 5G), showing that the point mutation did not affect transcription of *eg1*. As a control, no signal was detected when sense RNA was used as a probe (Figure 5H). Thus *EG1* is first strongly expressed in inflorescence meristems, and, with the transition from spikelet meristem to floral meristem and subsequently maturation of the flower, its expression decreases gradually, consistent with a role in early flower development.

### *OsLHS1* expression in the *eg1* mutants

**Figure 6:**
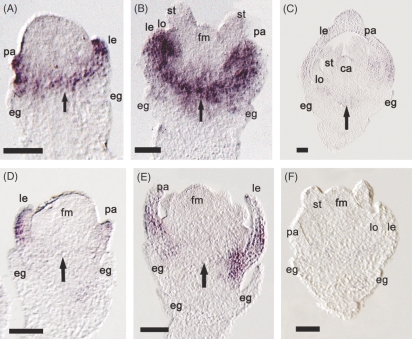
*In situ* hybridization of *OsLHS1* transcripts in wild-type and *eg1-1* flowers. (A)–(C) Wild-type flowers. (D, E) *eg1-1* flowers. *OsLHS1* transcripts are detected predominantly in palea and lemma primordium and inner zones (arrow) in wild-type flowers, but are absent in this region (arrow) in *eg1-1* flowers. (F) Hybridization with the *OsLHS1* sense probe. Scale bars = 50 μm (A, B, D, E) or 20 μm (C, F). ca, carpel; eg, empty glume; le, lemma; lo, lodicule; pa, palea; st, stamen.

The pleiotropic abnormalities of flower development observed in the *eg1* mutants suggested that they may result from alteration of expression patterns of genes involved in rice flower development. *OsLHS1* plays a role in palea and lemma identity specification and meristem determinacy, and mutations in *OsLHS1* affected all four floral whorls, with a floral phenotype highly similar to that of the *eg1* mutants (Jeon *et al.*, 2000; Prasad *et al.*, 2001, 2005). To investigate whether there was any relationship between *EG1* and *OsLHS1*, we examined the expression pattern of *OsLHS1* in the *eg1* mutant by RNA *in situ* hybridization. As reported previously (Prasad *et al.*, 2005), early expression of *OsLHS1* in the wild-type floral meristem was confined to the lemma and palea primordia at about the stage of their initiation (Figure 6A), and was barely detectable in the lodicules and stamen primordia where organ differentiation occurs (Figure 6B,C). Notably, *OsLHS1* RNA was uniformly detected in the central zone of flowers, resulting in a half ring-shaped signal (Figure 6A–C). The expression pattern in the *eg1-1* mutant was clearly distinguishable from that in the wild-type in that the *OsLHS1* signal was no longer detected in the inner zone of flowers, although *OsLHS1* RNA still appeared in the palea and lemma primordia (Figure 6D,E). These results suggest that *EG1* is required to maintain the inner zone expression of *OsLHS1* in floral meristems.

## Discussion

In this study, we have characterized a novel putative TAG lipase gene involved in rice spikelet development and specification of empty-glume identity. *EG1* appears to mediate expression of the floral meristem and organ identity gene *OsLHS1.* Our findings reveal a new major regulator in the regulation of rice spikelet development.

### EG1 is a major regulator of empty-glume fate

The glumes are a unique structure in grasses and account for some of the dramatic morphological variations in grass spikelets (Zanis, 2007). Our results show that *EG1* is a major regulator of empty-glume identity. The empty glumes are the most pronounced phenotype displayed by the *eg1* mutant. In both *eg1* mutant alleles, there were always some spikelets in which all floral organs appeared normal except for the empty glumes, especially in the weak allele *eg1-2* (Table 1). Approximately 30% of the *eg1-2* spikelets were normal with respect to the lemma, palea, lodicules and fertile organs, but the empty glumes were converted into glume-like organs or the number of empty glumes increased to three or four (Table 1). This phenotypic alteration could not be explained simply by down-regulation of *OsLHS1* in *eg1* mutants for the following reasons: first, in both wild-type and *eg1* mutants, expression of *OsLHS1* was completely absent in the empty glumes, whereas *EG1* is expressed strongly in empty glumes. Second, the *lhs1* mutant and plants with knockdown of *OsLHS1* by RNA interference both had normal empty glumes, although their inner floral organs exhibited severe defects (Jeon *et al.*, 2000). Thus, our results reveal that, in addition to acting as a regulator of floral meristem and organ identity genes (see below), an additional function of *EG1* is to specify empty-glume fate.

The identities of empty glumes and rudimentary glumes have been a subject of discussion, and two popular interpretations have been proposed for their origins and evolution (reviewed by Takeoka *et al.*, 1993). A widely accepted view is that the empty glumes are parts of a spikelet, and rudimentary glumes are bracts subtending them (Bell, 1991; Hoshikawa, 1989; Takeoka *et al.*, 1993). An alternative interpretation proposes that the empty glumes are two lower florets that have been reduced to sterile lemma during the course of evolution, and that the rudimentary glumes are the equivalents of glumes as is the case in other grass species (reviewed by Takeoka *et al.*, 1993). The *eg1* mutants exhibit pleiotropic floral defects in terms of organ identity, organ positioning, change in organ number and loss of floral meristem determinacy, and the rudimentary glumes are the only organs that are unaffected in the *eg1* spikelet. *EG1* transcipts first appear in the inflorescence meristem and then in the rudimentary glume and empty glume (Figure 5). However, we did not observe any abnormality in inflorescence or rudimentary-glume development. These results, together with observations in the *fzp* mutant, in which floral meristems were replaced by higher-order branches comprising several rudimentary glumes, and the results from overexpression of *OsLHS1* in rice, support the notion that empty glumes correspond to sterile lemmas, whereas the rudimentary glumes are equivalent to the glumes of other grass species (Arber, 1934; Komatsu *et al.*, 2003; Prasad *et al.*, 2001).

In addition, Whipple *et al.* (2007) investigated the relationship of lodicules to sterile floral organs of non-grass monocots by examining the expression of B-class genes. Their results support a conserved role for B-function genes across the angiosperms and additional evidence linking the evolution of lodicules and second-whorl tepals/petals of monocots. It is expected that further studies on *EG1* will provide important information on the molecular basis of spikelet variation and diversity in grasses as well as across the angiosperms.

### EG1 positively regulates expression of the floral meristem and organ identity gene *OsLHS1*

The similarities between the *eg1* mutants (Figure 1) and *lhs1* and *OsLHS1*-RNAi transgenic plants (Jeon *et al.*, 2000; Prasad *et al.*, 2005) indicated that they both function in spikelet development. However, their specific roles appear to be different. Empty glumes were not altered in the *lhs1* mutant or in *35sRNAi*-*OsLHS1* transgenic plants, but alterations in the number of empty glumes and homeotic transformation thereof were the most common phenotypes in *eg1* mutants. Another notable difference was that *OsLHS1* transcripts first appeared in the lemma primordium, and subsequently in palea and weakly in carpels, but were completely excluded from empty glumes and inflorescence meristems (Prasad *et al.*, 2001,^2005^). Also, *EG1* is expressed earlier than *OsLHS1*. *EG1* is strongly expressed in inflorescence meristems and in nearly all floral organs including the empty glumes (Figure 5). Furthermore, RNA *in situ* hybridization of *OsLHS1* showed that *OsLHS1* expression and pattern was altered and down-regulated, respectively, by the mutant *EG1* gene (Figure 6). Thus, we suggest that *EG1* and *OsLHS1* could work together or in parallel to regulate flower development in rice, and the altered expression of *OsLHS1* probably results in the pleiotropic phenotypes exhibited in the *eg1* spikelets.

### EG1 represents a potential lipid signaling pathway mediating floral development

It is unclear how EG1 functions to regulate spikelet development. EG1 belongs to the class III lipase family whose functions have seldom been reported (Ishiguro *et al.*, 2001; Ryu, 2004). Triacylglycerols (TAGs) are an important reserve of carbon and energy in eukaryotes. It is possible that *EG1* may be involved in mobilizing lipid reserves to provide energy that is specifically required during reproductive development. Another intriguing hypothesis is that an EG1-related biochemical process may play a signaling role in spikelet development. Evidence has shown that fatty acids can modulate protein kinase activities in plants (Scherer, 1996). In addition, there are a number of fatty acid-derived signals in plants: jasmonic acid (JA), the traumatin family and related alkenals (Farmer, 1994; Ishiguro *et al.*, 2001). It has been shown that TFL1, a phosphatidylethanolamine-binding protein (PEBP) (Bradley *et al.*, 1997; Ohshima *et al.*, 1997), is a key signaling protein that controls shoot meristem identity by translocation to domains where its target is located, even though *TFL1* mRNA is not found in these domains (Conti and Bradley, 2007). This non-autonomous action also seems to occur for *EG1*. *EG1* appears to regulate expression of *OsLHS1*, but the expression patterns of *EG1* and *OsLHS1* are not coincident. *EG1* is barely detected in the inner zone of the floral meristem at the stage of lemma and palea initiation (Figure 5E). By contrast, strong expression of *OsLHS1* in this region resulted in a half ring-shaped signal (Figure 6A,B). However, in the *eg1* mutant, expression of the *OsLHS1* was apparently absent in the inner zone of the floral meristem. It is likely that *EG1* may recruit an upstream factor(s) that is expressed in the inflorescence meristem to coordinate spikelet development at early stage and regulates the expression of MADS box transcription factors. Furthermore, recent evidence has indicated that genes related to lipid metabolism are involved in floral development. *AtDAD1*, a class I phospholipase A1, catalyzes the initial step of JA biosynthesis and controls anther dehiscence, pollen maturation and flower opening (Ishiguro *et al.*, 2001), and is the direct target of *AG* in coordinating late stamen maturation (Ito *et al.*, 2007). *AtAIM1* is involved in β-oxidation of fatty acids, and its loss of function causes a disorganized inflorescence meristem, abnormal floral organ development and alters the fatty acid composition of the mature adult plant (Richmond and Bleecker, 1999). These results indicate that a lipid signaling pathway probably plays a key role in plant flower development. Exploration of the biochemical function of EG1 and identification of its potential targets will provide better insight into the role of EG1 in mediating flower development in grasses.

## Experimental procedures

### Plant materials

Two rice single-gene recessive mutants, *eg1-1* and *eg1-2*, with floral organ defects, were used in this study. *eg1-1* (the origin of which is unknown) was further introgressed into the background of *O. sativa* L. spp. *indica* Zhefu 802 (ZF802) through several backcrosses. *eg1-2* was isolated from an M_2_ population of *O. sativa* L. spp. *japonica* Zhonghua 11(ZH11) produced by γ-ray irradiation. Allelism tests indicated that *eg1-2* was an allele of *eg1-1.* ZF802 was used as the wild-type for comparison of phenotype variations, microscopic analysis and RNA *in situ* hybridization.

### Microscopic analysis

Rice flowers from young panicles were fixed in 2.5% glutaraldehyde for at least 16 h at 4°C. They were dehydrated using a graded ethanol series, and then embedded in Leica 7022 historesin (http://www.leica-microsystems.com/). Samples were sectioned to 4 μm, stained with 0.1% toluidine blue-O and observed under an Olympus AX-80 light microscope (http://www.olympus.co.jp/en/). For scanning electron microscopy, young panicles were fixed in 4% glutaraldehyde, rinsed three times with 0.1 M sodium phosphate buffer, and fixed overnight in 2% OsO4 at 4°C. After dehydration using a graded ethanol series and isoamyl acetate replacement, the samples were then dried, mounted on SEM stubs and coated with gold. Mounted specimens were observed using a Hitachi S-800 scanning electron microscope (http://www.hitachi.com/).

### Positional cloning of *EG1*

The *EG1* locus was previously mapped to the long arm of rice chromosome 1 (Yoshimura *et al.*, 1997). *eg1-1* was crossed with ZF802, and 713 F_2_ plants with the extra glume phenotype were selected and used as a mapping population. Using multiple SSR, STS and CAPS markers, the *EG1* locus was confined to a 31 kb region on PAC clone P0035F12. For complementation tests, a 5618 bp genomic DNA fragment isolated from BAC clone OSJNBa0089G14 containing the entire *EG1* gene coding region, the 804 bp upstream sequence and the 3506 bp downstream sequence was cloned into a binary vector and introduced into *eg 1-2* homozygotes by *Agrobacterium tumefaciens*-mediated transformation (Hiei *et al.*, 1994).

### RNA expression analysis

RNA from root, stem, leaf, inflorescence, young flowers and developing seed was isolated using an RNeasy kit (Qiagen, http://www.qiagen.com). Power SYBR Green PCR Master Mix (Applied Biosystems, http://www.appliedbiosystems.com/) was used for quantitative real-time PCR with the primers shown in Table S1. Amplification of 18S *rRNA* was used as an internal control to normalize all data.

*In situ* hybridization was performed as previously described (Lai *et al.*, 2002). A gene-specific region at the 3′ end of *EG1* and *OsLHS1* were amplified and cloned into a T-vector and used for synthesis of an RNA probe (Table S1). Shoot apices of rice seedlings at the three-leaf stage and floral primordia of young panicles were fixed using formalin/acetic acid/alcohol (FAA) fixative solution at 4°C overnight, dehydrated and embedded in Paraplast Plus paraffin (Sigma, http://www.sigmaaldrich.com/). Tissues were sliced into 8 μm sections and affixed to Poly-Prep slides (Sigma). Images were observed under an Olympus BX51 microscope, and photographed using a Micro Color CCD camera (Apogee Instruments Inc., http://www.ccd.com/).

## Supporting Information

**Table S1.** Primers used in this study.

**Figure S1.** Quantitative real-time PCR analysis of *EG1* expression in various plant tissues.
