# Supplementary material for: A putative lipase gene EXTRA GLUME1 regulates both empty-glume fate and spikelet development in rice
Source: Plant J. 2008 Nov 7;57(4):593–605. doi: 10.1111/j.1365-313X.2008.03710.x (PMC2667685; doi:10.1111/j.1365-313X.2008.03710.x)
Supplement: Supplementary file 1 [file tpj0057-0593-SD1.doc]

**Supplementary Table 1** Primers Used in this study

Gene name Sequence*EG1* (in situ)F 5' CGGCAACATGGCGAGGAA 3'

R 5' TTGCCTCGGGAGCGTCATT 3'*OsLHS1* (in situ)

*18sRNA (*qRT-PCR*)*

*EG1* (qRT-PCR)

F 5' CCAGTGCAGAGAATGTGCTCCA 3'

R 5'GAAGGTCGTAAGAGAGCACGC 3'

F 5' CGGCTACCACATCCAAGGAA 3'

R 5' TGTCACTACCTCCCCGTGTCA 3'

F 5' AACGTACACGACCCGATCAC 3'

R 5' GACGTGGGTGTAGCAGGAGT 3'
